# Supplementary material for: Small deviations in kinematics and body form dictate muscle performances in the finely tuned avian downstroke
Source: eLife. 2024 Feb 26;12:RP89968. doi: 10.7554/eLife.89968 (PMC10942624; doi:10.7554/eLife.89968)
Supplement: Supplementary file 1. — (a) Air conditions across all 20 flights. N = 4 doves; n = 5 flights each. (b) Measured aerodynamic and leg impulses before surgery, after surgery, and after surgery with the suspended recording cable (for recording EMG and sonomicrometry). So that horizontal and vertical impulses can be compared, gravity has been subtracted from the vertical impulse, so that while at rest, the vertical impulse is zero (horizontal and vertical impulse scaled by bodyweight, bw and integration time; bodyweight is based on the average vertical force on the takeoff perch before takeoff). These definitions for impulse match Chin and Lentink, 2017 paper on directional impulse of birds (Chin and Lentink, 2017). (c) Measured aerodynamic and leg impulses among individual doves. The definition for impulse matches Supplementary file 1b. (d) Measured aerodynamic forces for the second stroke after takeoff (Figure 3). The weight of the dove is primarily supported during the downstroke. Drag partially contributes to weight support, while opposing forward aerodynamic force. The reported values are the stroke-averaged aerodynamic force measured by the aerodynamic force platform (AFP), normalized by bodyweight. We compute the time-resolved lift and drag using the measured horizontal and vertical aerodynamic forces together with the measured wing velocity. N = 4 doves; n = 5 flights each; mean ± standard deviation. (e) We compute aerodynamic, inertial, and required muscle power based on first principals (Figure 4) and model the effect of the amount of energy storage in the supracoracoideus (Figure 5). The primary component of stroke-averaged net muscle power is aerodynamic, whereas the positive and negative regions of inertial power cancel. Our energy storage model predicts that the pectoralis would need to generate 24.9% ± 17.5% more power to fully power the upstroke via energy storage in the supracoracoideus. N = 4 doves; n = 5 flights each; mean ± standard deviation. [file elife-89968-supp1.pdf]

Supplementary File 1a. Air conditions across all 20 flights. (N = 4 doves; n = 5 flights each).

Variable	Mean ± s.d.
Air density [$kg \cdot m^{-3}$]	1.191 ± 0.001
Air temperature [°C]	23.73 ± 0.28
Air pressure [kPa]	101.78 ± 0.06
Air relative humidity [%]	27.05 ± 6.75

Supplementary File 1b. Measured aerodynamic and leg impulses before surgery, after surgery, and after surgery with the suspended recording cable (for recording EMG & sonomicrometry). So that horizontal and vertical impulses can be compared, gravity has been subtracted from the vertical impulse, so that while at rest, the vertical impulse is zero (horizontal and vertical impulse scaled by bodyweight, bw and integration time; bodyweight is based on the average vertical force on the takeoff perch before takeoff).

Variable	Before surgery	After surgery (no cable)	After surgery (with cable)
Total bodyweight measured by takeoff perch [g]	158.4 ± 0.8	159.1 ± 3.6	170.0 ± 1.1
Vertical takeoff impulse (perch) [-]	0.39 ± 0.03	0.34 ± 0.03	0.29 ± 0.05
Vertical aerodynamic impulse (all strokes) [-]	-0.22 ± 0.02	-0.28 ± 0.04	-0.23 ± 0.06
Vertical landing impulse (perch) [-]	0.09 ± 0.07	0.12 ± 0.15	-0.09 ± 0.13
Vertical total impulse [-]	0.03 ± 0.01	0.03 ± 0.03	0.01 ± 0.01
Vertical aerodynamic impulse (stroke 2) [-]	0.06 ± 0.04	-0.22 ± 0.03	-0.17 ± 0.06
Horizontal takeoff impulse (perch) [-]	0.81 ± 0.03	0.56 ± 0.05	0.64 ± 0.03
Horizontal aerodynamic impulse (all strokes) [-]	-0.09 ± 0.02	-0.03 ± 0.01	0.02 ± 0.03
Horizontal landing impulse (perch) [-]	-0.35 ± 0.04	-0.43 ± 0.14	-0.42 ± 0.19
Horizontal total impulse [-]	0.03 ± 0.03	0.01 ± 0.01	0.08 ± 0.01
Horizontal aerodynamic impulse (stroke 2) [-]	-0.11 ± 0.05	0.08 ± 0.04	0.15 ± 0.02

Supplementary File 1c. Measured aerodynamic and leg impulses among individual doves. The definition for impulse matches Supplementary File 1b.

Variable	Dove 1	Dove 2	Dove 3	Dove 4	All doves
Total bodyweight measured by takeoff perch [g]	152.6 ± 0.9	166.7 ± 3.4	170.0 ± 1.1	182.3 ± 1.7	167.9 ± 11.0
Vertical takeoff impulse (perch) [-]	0.34 ± 0.04	0.26 ± 0.11	0.29 ± 0.05	0.23 ± 0.06	0.28 ± 0.08
Vertical aerodynamic impulse (all strokes) [-]	-0.19 ± 0.03	-0.26 ± 0.09	-0.23 ± 0.06	-0.18 ± 0.06	-0.22 ± 0.07
Vertical landing impulse (perch) [-]	-0.09 ± 0.05	-0.25 ± 0.16	-0.09 ± 0.13	-0.16± 0.11	-0.15 ± 0.13
Vertical total impulse [-]	0.00 ± 0.01	-0.01 ± 0.02	0.01 ± 0.01	0.02 ± 0.01	0.01 ± 0.02
Vertical aerodynamic impulse (stroke 2) [-]	-0.05 ± 0.05	-0.21 ± 0.09	-0.17 ± 0.06	-0.13 ± 0.06	-0.14 ± 0.09
Horizontal takeoff impulse (perch) [-]	0.77 ± 0.06	0.69 ± 0.03	0.64 ± 0.03	0.44 ± 0.04	0.63 ± 0.13
Horizontal aerodynamic impulse (all strokes) [-]	-0.09 ± 0.02	-0.01 ± 0.02	0.02 ± 0.03	0.05 ± 0.02	-0.01 ± 0.06
Horizontal landing impulse (perch) [-]	-0.29 ± 0.02	-0.12 ± 0.13	-0.42 ± 0.19	-0.34 ± 0.14	-0.29 ± 0.16
Horizontal total impulse [-]	0.08 ± 0.03	0.10 ± 0.01	0.08 ± 0.01	0.08 ± 0.01	0.08 ± 0.02
Horizontal aerodynamic impulse (stroke 2) [-]	-0.09 ± 0.01	-0.03 ± 0.09	0.15 ± 0.02	0.17 ± 0.03	0.05 ± 0.13

Supplementary File 1d. Measured aerodynamic forces for the second stroke after takeoff (Figure 3). The weight of the dove is primarily supported during the downstroke. Drag partially contributes to weight support, while opposing forward aerodynamic force. The reported values are the stroke-averaged aerodynamic force measured by the Aerodynamic Force Platform (AFP), normalized by bodyweight. We compute the time-resolved lift and drag using the measured horizontal and vertical aerodynamic forces together with the measured wing velocity. (*N* = 4 doves; *n* = 5 flights each; mean ± standard deviation).

Stroke-averaged aerodynamic force / bodyweight [%]	Entire stroke	Downstroke	Upstroke	Lift	Drag
Weight support (vertical force)	87.28 ± 9.94	76.00 ± 7.60	11.28 ± 2.81	66.17 ± 10.81	21.11 ± 4.62
Horizontal force	5.21 ± 12.60	2.52 ± 11.74	2.68 ± 1.49	32.84 ± 9.96	-27.64 ± 4.64

Supplementary File 1e. We compute aerodynamic, inertial, and required muscle power based on first principals (Figure 4), and model the effect of the amount of energy storage in the supracoracoideus (Figure 5). The primary component of stroke-averaged net muscle power is aerodynamic, whereas the positive and negative regions of inertial power cancel. Our energy storage model predicts that the pectoralis would need to generate 24.9% ± 17.5% more power to fully power the upstroke via energy storage in the supracoracoideus. (*N* = 4 doves; *n* = 5 flights each; mean ± standard deviation).

Stroke-averaged power / pectoralis mass [W/kg]	Aerodynamic	Inertial	Muscle	Pectoralis: no energy storage	Pectoralis: 100% energy storage
Net power	210.0 ± 56.9	-1.4 ± 20.0	207.6 ± 56.6	181.8 ± 47.6	231.7 ± 59.6
Positive power	222.4 ± 51.5	80.0 ± 21.9	249.3 ± 59.8	206.0 ± 49.6	255.9 ± 62.7
Negative power	-13.5 ± 10.3	-81.4 ± 22.8	-41.7 ± 22.2	-24.2 ± 15.3	-24.2 ± 15.3
Max power	775.3 ± 179.0	348.0 ± 112.5	711.3 ± 150.7	734.5 ± 165.5	varies
