# Supplementary material for: Small deviations in kinematics and body form dictate muscle performances in the finely tuned avian downstroke
Source: eLife. 2024 Feb 26;12:RP89968. doi: 10.7554/eLife.89968 (PMC10942624; doi:10.7554/eLife.89968)
Supplement: Supplementary file 2. — (a) Scaling analysis (Figure 7) parameter summary. (b) Scaling analysis parameters used for analyzing shifts in the timing of the pectoralis power distribution during the stoke (Figure 7D). [file elife-89968-supp2.pdf]

Supplementary File 2a. Scaling analysis (Figure 7) parameter summary.

Variable	Scaling equation	Description	Method for acquiring
m_{body}		Body mass	Measured extant bird data from literature (31 birds)
m_{pect}		Pectoralis mass (single pectoralis)	Measured extant bird data from literature scaled isometrically to match body mass (exact match: 25 birds, close relative: 6 birds)
m_{wing}		Wing mass (single wing)	Measured extant bird data from literature (31 birds)
I_{wing}		Wing moment of inertia (single wing)	Measured extant bird data from literature (27 birds) or determined based on scaling law reported in literature: Berg & Rayner 1995 (close relative: 4 birds)
$\lvert\vec{\mathbf{r}}_{\text{span}}\rvert$		Wingspan (distance from shoulder joint to wingtip; single wing)	Measured extant bird data from literature (27 birds) or measured extant bird data from literature scaled isometrically to match body mass (close relative: 4 birds)
$\lvert\vec{\mathbf{r}}_{\text{wing,cg}}\rvert$		Distance from shoulder joint to center of gravity of wing (single wing)	Measured extant bird data from literature (27 birds) or determined isometrically to match body mass (close relative: 4 birds)
S_{wing}		Wing area (single wing)	Measured extant bird data from literature (27 birds) or measured extant bird data

			from literature scaled isometrically to match body mass (close relative: 4 birds)
f		Wingbeat frequency	Measured extant bird data from literature (21 birds) or determined based on scaling law reported in literature: Berg & Rayner 1995 (10 birds)
$m_{\text{wing,i}}$		Wing mass distribution (point masses)	Least-squares fit to match wing mass, inertia, and center of gravity data
Δt	$\propto f^{-1}$	Time step	Assumed to scale isometrically
$\vec{\mathbf{F}}_{\text{aero}}$	$\propto m_{\text{body}}$	Aerodynamic force	Assumed to scale isometrically
${}^{\text{N}}\vec{\mathbf{v}}^{\text{i}}_{\text{wing}}$	$\propto f\left\|\vec{\mathbf{r}}_{\text{span}}\right\|$	Wing velocity (distributed)	Assumed to scale isometrically
${}^{\text{N}}\vec{\mathbf{v}}_{\text{body}}$	$\propto 1$	Body velocity	Unchanged
$\chi_{\text{p,aero}}$	$=\left\|\vec{r}_{span}\right\| f$	Scaling parameter which dictates the aerodynamic power contribution ∝ wingtip speed	Derived (Eqn. S71)
$\chi_{\text{m,aero}}$	$=\left\|\vec{r}_{span}\right\|$	Scaling parameter which dictates the aerodynamic moment contribution = wingspan	Derived (Eqn. S72)
$\chi_{\text{p,iner}}$	$=\frac{I_{\text{wing}}}{m_{\text{body}}} f^3$ $=\frac{m_{\text{wing}}}{m_{\text{body}}}\left(\frac{r_{\text{gyr}}}{\left\|\vec{\mathbf{r}}_{\text{span}}\right\|}\right)^2\left(f\left\|\vec{\mathbf{r}}_{\text{span}}\right\|\right)^2 f$	Scaling parameter which dictates the inertial power contribution ∝ (wing mass %) (wing radius of gyration %)^2 (wingtip speed)^2 (wingbeat frequency)	Derived (Eqn. S73)

$\chi_{\mathrm{m,iner}}$	$= \frac{I_{\mathrm{wing}}}{m_{\mathrm{body}}} f^2 = \frac{m_{\mathrm{wing}}}{m_{\mathrm{body}}} \left(\frac{r_{\mathrm{gyr}}}{\lvert \vec{\mathbf{r}}_{\mathrm{span}} \rvert} \right)^2 \left(f \lvert \vec{\mathbf{r}}_{\mathrm{span}} \rvert \right)^2$	Scaling parameter which dictates the inertial moment contribution ∝ (wing mass %) (wing radius of gyration %)^2 (wingtip speed)^2	Derived (Eqn. S74)

Supplementary File 2b. Scaling analysis parameters used for analyzing shifts in the timing of the pectoralis power distribution during the stoke (Figure 7D).

Variable	Scaling equation	Description	Method for acquiring
m_{body}		Body mass	Measured extant bird data from literature (17 birds)
m_{wing}		Wing mass (single wing)	Measured extant bird data from literature (1 birds) or measured extant bird data from literature scaled isometrically to match body mass (16 birds)
$\left\|\vec{\mathbf{r}}_{\text{span}}\right\|$		Wingspan (distance from shoulder joint to wingtip; single wing)	Measured extant bird data from literature (3 birds) or measured extant bird data from literature scaled isometrically to match body mass (14 birds)
f		Wingbeat frequency	Measured extant bird data from literature (17 birds)
EMG_{start}		Start of electrical activation of pectoralis (stroke %)	Measured extant bird data from literature (17 birds)
EMG_{end}		End of electrical activation of pectoralis (stroke %)	Measured extant bird data from literature (17 birds)
$F_{pect,max}$		Max force generated by pectoralis (stroke %)	Measured extant bird data from literature (12 birds)
I_{wing}		Wing moment of inertia (single wing)	Determined based on scaling law reported in literature: Berg & Rayner 1995 (17 birds)

$\chi_{\text{p,iner}} / \chi_{\text{p,aero}}$	$= x_{\text{m,iner}} / x_{\text{m,aero}}$ $= \dfrac{I_{\text{wing}}}{m_{\text{body}} \lvert \vec{r}_{span} \rvert} f^{2}$ $= \dfrac{m_{\text{wing}}}{m_{\text{body}}} \left(\dfrac{r_{\text{gyr}}}{\lvert \vec{\mathbf{r}}_{\text{span}} \rvert} \right)^{2} \left(f \lvert \vec{\mathbf{r}}_{\text{span}} \rvert \right) f$	Scaling parameter which dictates the aerodynamic power contribution ∝ wingtip speed	Derived
