# Supplementary material for: Small deviations in kinematics and body form dictate muscle performances in the finely tuned avian downstroke
Source: eLife. 2024 Feb 26;12:RP89968. doi: 10.7554/eLife.89968 (PMC10942624; doi:10.7554/eLife.89968)
Supplement: Supplementary file 3. — (a) Detailed breakdown of data from extant birds used for the scaling analysis (Figure 7). Some data was scaled isometrically by bodyweight based on matching the species of the bird (blue). Other data was found based on published scaling relationships (green). The references corresponding to the letter plus number keys (found in columns titled Src) are in Supplementary file 3c. To match some of the bird species, a close relative was used when data was missing for the original species: close relative of Parus ater used was Parus cristatus mitratus. Close relative of Perisoreus canadensis used was Xanthoura yncas. Close relative of Pica hudsonia used was Pica pica. Close relative of Corvus brachyrhynchos used was Corvus vorone. Close relative of Alectoris rufa used was Coturnix. Close relative of Corvus corax used was Corvus vornix. (b) Detailed breakdown of data from extant birds used for analyzing shifts in the timing of the pectoralis power distribution during the stoke for the scaling analysis (Figure 7D). The same color scheme is used as in Supplementary file 3a, and the close relative species used when data was missing for the original species are: close relative of Selasphorus rufus used was Calypte anna. Close relative of Perisoreus canadensis used was Xanthoura yncas. Close relative of Pica hudsonia used was Pica pica. Close relative of Corvus brachyrhynchos used was Corvus corone. Close relative of Alectoris chukar used was Caccabis rufa. Close relative of Corvus corax used was Corvus cornix. (c) Keys for references in Supplementary files 3a and b. [file elife-89968-supp3.pdf]

Supplementary File 3a. Detailed breakdown of data from extant birds used for the scaling analysis (Figure 7). Some data was scaled isometrically by bodyweight based on matching the species of the bird (blue). Other data was found based on published scaling relationships (green). The references corresponding to the letter plus number keys (found in columns titled Src) are in Supplementary File 3c. To match some of the bird species, a close relative was used when data was missing for the original species: Close relative of Parus ater used was Parus cristatus mitratus. Close relative of Perisoreus canadensis used was Xanthoura yncas. Close relative of Pica hudsonia used was Pica pica. Close relative of Corvus brachyrhynchos used was Corvus vorone. Close relative of Alectoris rufa used was Coturnix. Close relative of Corvus corax used was Corvus vornix.

Bird		Body mass [g]		Single pectoralis mass [g]		Single wing mass [g]		Wingspan: shoulder joint to wingtip [mm]		Center of gravity dist. to shoulder joint [mm]		Single wing moment of inertia [$10^6 \cdot kg \cdot m^2$]		Wing area [cm^2]		Wingbeat frequency [Hz]	
Scientific name	Common name	#	Src	#	Src	#	Src	#	Src	#	Src	#	Src	#	Src	#	Src
Parus caeruleus	Blue tit	9.5	B95	0.764	G62	0.48	B95	95	B95	18	B95	0.286	B95	42.4	B95	21.7	B95
Poephila guttata	Zebra finch	14.5	B95	0.98	T05	0.63	B95	90	B95	19	B95	0.356	B95	33	B95	27.4	B95
Parus ater	Coal tit	14.6	B95	1.016	G62	0.84	B95	112	B95	21	B95	0.673	B95	51.9	B95	18.2	B95
Erithacus rubecula	Robin	15	B95	0.904	G62	0.69	B95	108	B95	22	B95	0.581	B95	46.5	B95	16.1	B95
Delichon urbica	House martin	15.2	B95	1.006	G62	0.85	B95	140	B95	30	B95	1.348	B95	51.5	B95	9.4	B95
Fringilla coelebs	Chaffinch	19.9	B95	2.33	G62	1.1	B95	116	B95	23	B95	1.007	B95	55.8	B95	17.3	B95

Bird		Body mass [g]		Single pectoralis mass [g]		Single wing mass [g]		Wingspan: shoulder joint to wingtip [mm]		Center of gravity dist. to shoulder joint [mm]		Single wing moment of inertia [10^6·kg·m^2]		Wing area [cm^2]		Wingbeat frequency [Hz]	
Scientific name	Common name	#	Src	#	Src	#	Src	#	Src	#	Src	#	Src	#	Src	#	Src
Alcedo atthis	Common kingfisher	34.3	B95	2.836	G62	1.55	B95	130	B95	30	B95	2.344	B95	62	B95	15.6	B95
Turdus merula	Blackbird	56.2	B95	4.48	G62	3.14	B95	184	B95	42	B95	9.115	B95	127.8	B95	8.6	B95
Sturnus vulgaris	European starling	58.1	B95	5.48	G62	3.7	B95	185	B95	41	B95	10.16	B95	120.8	B95	11.3	B95
Picoides major	Great spotted woodpecker	62.9	B95	5.6	G62	4.17	B95	190	B95	42	B95	11.15	B95	121.8	B95	10.4	B95
Turdus philomelos	Song thrush	67.6	B95	6.73	G62	3.39	B95	171	B95	33	B95	6.098	B95	103.5	B95	10.0	B95
Perisoreus canadensis	Gray jay	68.9	J11A	4.5	G62	4.48	G62	172	G62	36	B95	10.89	B95	154	G62	11.6	J11A
Alle alle	Dovekie (little auk)	100	B95	7.02	G62	5.04	B95	183	B95	46	B95	16.18	B95	65	B95	9.4	B95
Coturnix coturnix	Quail	132	B95	11.7	G62	4	B95	164	B95	37	B95	8.482	B95	91.8	B95	17.8	B95
Podiceps ruficollis	Little grebe	146	B95	5.94	G62	4.56	B95	194	B95	53	B95	20.03	B95	110.1	B95	10.2	B95

Bird		Body mass [g]		Single pectoralis mass [g]		Single wing mass [g]		Wingspan: shoulder joint to wingtip [mm]		Center of gravity dist. to shoulder joint [mm]		Single wing moment of inertia $[10^{6} \cdot kg \cdot m^{2}]$		Wing area $[cm^{2}]$		Wingbeat frequency [Hz]	
Scientific name	Common name	#	Src	#	Src	#	Src	#	Src	#	Src	#	Src	#	Src	#	Src
Streptopelia risoria	Ringneck dove	161.6	CURR	14.66	CURR	8.62	CURR	217	CURR	52	CURR	35.3	CURR	182.6	CURR	9.8	CURR
Scolopax rusticola	Woodcock	163	B95	20.75	G62	12.45	B95	279	B95	68	B95	84.98	B95	254.2	B95	6.9	B95
Pica hudsonia	Black-billed magpie	172.4	J11A	11	G62	12.6	G62	218	G62	46	B95	52.67	B95	277	G62	9.0	J11A
Picus viridis	Green woodpecker	179	B95	14.57	G62	11.75	B95	235	B95	51	B95	47.54	B95	231	B95	8.3	B95
Larus ridibundus	Black-headed gull	256	B95	16.33	G62	18.98	B95	461	B95	118	B95	402.5	B95	442.2	B95	3.5	B95
Accipiter nisus	Eurasian sparrowhawk	279	B95	28.47	G62	22.61	B95	355	B95	93	B95	263	B95	377	B95	4.5	B95
Columba livia	Rock pigeon	293	B95	27.63	T08	22.47	B95	323	B95	71	B95	172.7	B95	314	B95	6.7	B95
Corvus frugilegus	Rook	328	B95	24.15	G62	28.78	B95	389	B95	80	B95	326.9	B95	419.8	B95	4.0	B95
Gallinula chloropus	Grey moorhen	364	B95	22.66	G62	14.2	B95	271	B95	65	B95	95.58	B95	254.9	B95	7.2	B95

Bird		Body mass [g]		Single pectoralis mass [g]		Single wing mass [g]		Wingspan: shoulder joint to wingtip [mm]		Center of gravity dist. to shoulder joint [mm]		Single wing moment of inertia [$10^6 \cdot kg \cdot m^2$]		Wing area [cm^2]		Wingbeat frequency [Hz]	
Scientific name	Common name	#	Src	#	Src	#	Src	#	Src	#	Src	#	Src	#	Src	#	Src
Corvus brachyrhynchos	American crow	387.6	J11A	27.6	G62	30.8	G62	302	G62	63	B95	261.3	B95	465	G62	6.4	J11A
Alectoris rufa	Red-legged partridge	393	B95	39.3	G62	19.15	B95	254	B95	60	B95	100.7	B95	241.1	B95	7.7	B95
Strix aluco	Tawny owl	398	B95	18.81	G62	31.41	B95	421	B95	111	B95	578.5	B95	617.7	B95	4.5	B95
Melanitta nigra	Common scoter	569	B95	33.36	G62	38.85	B95	390	B95	108	B95	656.6	B95	422.6	B95	4.9	B95
Uria aalge	Common murre (guillemot)	691	B95	50.63	G62	31.07	B95	335	B95	100	B95	424.8	B95	241.6	B95	9.4	B95
Buteo buteo	Common buzzard	771	B95	42.6	G62	72.51	B95	590	B95	155	B95	2536	B95	1142	B95	3.2	B95
Corvus corax	Common raven	894.9	J11A	65.6	G62	67.9	G62	342	G62	72	B95	773.6	B95	829	G62	5.6	J11A
Ardea cinerea	Grey heron	2140	B95	164.9	G62	167.1	B95	830	B95	216	B95	12334	B95	1997	B95	2.6	B95

Supplementary File 3b. Detailed breakdown of data from extant birds used for analyzing shifts in the timing of the pectoralis power distribution during the stoke for the scaling analysis (Figure 7D). The same color scheme is used as in Supplementary File 3a, and the close relative species used when data was missing for the original species are: Close relative of Selasphorus rufus used was Calypte anna. Close relative of Perisoreus canadensis used was Xanthoura yncas. Close relative of Pica hudsonia used was Pica pica. Close relative of Corvus brachyrhynchos used was Corvus corone. Close relative of Alectoris chukar used was Caccabis rufa. Close relative of Corvus corax used was Corvus cornix.

Bird		Flight mode	Body mass [g]		Single wing mass [g]		Wingspan: shoulder joint to wingtip [mm]		Single wing moment of inertia [$10^{6}\cdot kg\cdot m^{2}$]		Wingbeat frequency [Hz]		Pectoralis EMG start & end time [stroke %]			Max pectoralis force time [stroke %]	
Scientific name	Common name		#	Src	#	Src	#	Src	#	Src	#	Src	Start	End	Src	#	Src
Selasphorus rufus	Rufous Hummingbird	0-10 m/s	3.4	T10	0.084	I18	45.5	I18	0.0110	B95	42	T10	-29.3	-8.8	T10		
Calypte anna	Anna's hummingbird	Hovering	4.2	D13	0.104	I18	48.8	I18	0.0159	B95	40	D13	-22.9	-14.2	D13		
Calypte anna	Anna's hummingbird	Hovering	4.9	I18	0.121	I18	51.4	I18	0.0225	I18	41	I18	-25.4	-16.0	A10	4.2	A10
Taeniopygia guttata	Zebra finch		11.1	D13	0.48	B95	82.3	B95	0.231	B95	27.1	D13	-16.5	3.0	D13		
Taeniopygia guttata	Zebra finch	Ave: ascend, 0, 2, 4 m/s	13.3	T05	0.58	B95	72	T05	0.213	B95	27	T05	-14.5	27.7	T05		
Perisoreus canadensis	Gray jay	Takeoff	68.9	J11A	4.48	G62	172	G62	10.89	B95	11.6	J11A	-9.7	30.8	J11A	30.5	J11A

Bird		Flight mode	Body mass [g]		Single wing mass [g]		Wingspan: shoulder joint to wingtip [mm]		Single wing moment of inertia [$10^6 \cdot kg \cdot m^2$]		Wingbeat frequency [Hz]		Pectoralis EMG start & end time [stroke %]			Max pectoralis force time [stroke %]	
Scientific name	Common name		#	Src	#	Src	#	Src	#	Src	#	Src	Start	End	Src	#	Src
Sturnus vulgaris	European starling	13.7 m/s	71.7	B92	4.55	B95	198	B95	14.97	B95	15	B92	-19.4	20.9	B92	28.4	B92
Nymphicus hollandicus	Cockatiel	7 m/s	85	H03	8.1	H04	232	H04	37.72	B95	7.64	H03	-27.9	17.1	H03	13.0	H03
Pica hudsonia	Black-billed magpie	Takeoff	172.4	J11A	12.6	G62	218	G62	52.67	B95	9	J11A	-9.4	28.2	J11A	14.0	J11A
Pica pica	Magpie	6 m/s	174	D97	12.7	G62	219	G62	53.45	B95	8.5	D97	-35.3	9.3	D97	15.8	D97
Columba livia	Pigeon	Takeoff	309	D93	23.6	B95	329	B95	237.1	B95	9	D93	-28.1	27.2	D93	16.5	D93
Corvus brachyrhynchos	American crow	Takeoff	387.6	J11A	30.8	G62	302	G62	261.3	B95	6.4	J11A	-2.7	39.7	J11A	27.4	J11A
Columba livia	Rock pigeon	Level flight	441.4	J11B	33.8	B95	370	B95	441.1	B95	8.9	J11B	-17.4	26.7	J11B	26.9	J11B
Columba livia	Rock pigeon	Ascending flight	452	J11B	34.6	B95	373	B95	459.4	B95	8.9	J11B	-18.9	26.1	J11B	26.5	J11B
Alectoris chukar	Chukar	Adult flight	549	T17	23.9	G62	172	G62	62.72	B95	22	T17	-12.8	14.5	T17		
Columba livia	Rock pigeon	0-2 m/s	562	T08	43	B95	330	T08	445.9	B95	8.6	T08	-11.8	30.0	T08	24.3	T08

Bird		Flight mode	Body mass [g]		Single wing mass [g]		Wingspan: shoulder joint to wingtip [mm]		Single wing moment of inertia [$10^6 \cdot kg \cdot m^2$]		Wingbeat frequency [Hz]		Pectoralis EMG start & end time [stroke %]			Max pectoralis force time [stroke %]	
Scientific name	Common name		#	Src	#	Src	#	Src	#	Src	#	Src	Start	End	Src	#	Src
Corvus corax	Common raven	Takeoff	894.9	J11A	67.9	G62	342	G62	773.6	B95	5.6	J11A	-6.1	46.0	J11A	48.2	J11A

Supplementary File 3c. Keys for references in Supplementary Files 3a and 3b.

Abbrev	Full source
CURR	Current work
A10	[1]
B95	[2]
B92	[3]
D93	[4]
D97	[5]
D13	[6]
G62	[7]
H03	[8]
H04	[9]
I18	[10]
J11A	[11]
J11B	[12]
T05	[13]
T08	[14]
T10	[15]
T17	[16]

References

[1] D. L. Altshuler *et al.*, “Neuromuscular control of wingbeat kinematics in Anna’s hummingbirds (Calypte anna),” *Journal of Experimental Biology*, vol. 213, no. 14, pp. 2507–2514, 2010, doi: 10.1242/jeb.043497.

[2] C. Van Den Berg and J. M. V. Rayner, “The moment of inertia of bird wings and the inertial power requirement for flapping flight,” *J Exp Biol*, vol. 198, pp. 1655–64, 1995.

[3] A. A. Biewener, K. P. Dial, and G. E. Goslow Jr., “Pectoralis Muscle Force and Power Output During Flight in the Starling,” *Journal of Experimental Biology*, vol. 164, no. 1, pp. 1–18, 1992.

[4] K. P. Dial and A. A. Biewener, “Pectoralis Muscle Force and Power Output During Different Modes of Flight in Pigeons,” *J Exp Biol*, vol. 176, pp. 31–54, 1993.

[5] K. P. Dial, A. A. Biewener, B. W. Tobalske, and D. R. Warrick, “Mechanical power output of bird flight,” *Nature*, vol. 390, no. 6655, pp. 67–70, 1997, doi: 10.1038/36330.

[6] E. R. Donovan, B. K. Keeney, E. Kung, S. Makan, J. M. Wild, and D. L. Altshuler, “Muscle Activation Patterns and Motor Anatomy of Anna’s Hummingbirds Calypte anna and Zebra Finches Taeniopygia guttata,” *Physiological and Biochemical Zoology*, vol. 86, no. 1, pp. 27–46, 2013, doi: 10.1086/668697.

[7] C. H. Greenewalt, “Dimensional relations for flying animals,” *Smithsonian Miscellaneous Collections*, vol. 144, no. 2, pp. 1–46, 1962, doi: 10.4324/9780203476154_chapter_13.

[8] T. L. Hedrick, B. W. Tobalske, and A. A. Biewener, “How cockatiels (Nymphicus hollandicus) modulate pectoralis power output across flight speeds,” *J Exp Biol*, vol. 206, no. 8, pp. 1363–1378, 2003, doi: 10.1242/jeb.00272.

[9] T. L. Hedrick, J. R. Usherwood, and A. A. Biewener, “Wing inertia and whole-body acceleration: an analysis of instantaneous aerodynamic force production in cockatiels (Nymphicus hollandicus) flying across a range of speeds,” *J Exp Biol*, vol. 207, no. 10, pp. 1689–1702, 2004, doi: 10.1242/jeb.00933.

[10] R. Ingersoll and D. Lentink, “How the hummingbird wingbeat is tuned for efficient hovering,” *J Exp Biol*, vol. 221, 2018, doi: 10.1242/jeb.178228.

[11] B. E. Jackson and K. P. Dial, “Scaling of mechanical power output during burst escape flight in the Corvidae,” *Journal of Experimental Biology*, vol. 214, no. 3, pp. 452–461, 2011, doi: 10.1242/jeb.046789.

[12] B. E. Jackson, B. W. Tobalske, and K. P. Dial, “The broad range of contractile behaviour of the avian pectoralis: functional and evolutionary implications,” *Journal of Experimental Biology*, vol. 214, no. 14, pp. 2354–2361, 2011, doi: 10.1242/jeb.052829.

[13] B. W. Tobalske, L. A. Puccinelli, and D. C. Sheridan, “Contractile activity of the pectoralis in the zebra finch according to mode and velocity of flap-bounding flight,” *J Exp Biol*, vol. 208, no. 15, pp. 2895–2901, 2005, doi: 10.1242/jeb.01734.

[14] B. W. Tobalske and A. A. Biewener, “Contractile properties of the pigeon supracoracoideus during different modes of flight,” *Journal of Experimental Biology*, vol. 211, no. 2, pp. 170–179, 2008, doi: 10.1242/jeb.007476.

[15] B. W. Tobalske, A. A. Biewener, D. R. Warrick, T. L. Hedrick, and D. R. Powers, “Effects of flight speed upon muscle activity in hummingbirds,” *Journal of Experimental Biology*, vol. 213, no. 14, pp. 2515–2523, 2010, doi: 10.1242/jeb.043844.

[16] B. W. Tobalske, B. E. Jackson, and K. P. Dial, “Ontogeny of flight capacity and pectoralis function in a precocial ground bird (alectoris chukar),” *Integr Comp Biol*, vol. 57, no. 2, pp. 217–230, 2017, doi: 10.1093/icb/icx050.
